# HPCAL1 promotes colorectal cancer progression via TCF7/p65-mediated Wnt ligand upregulation and Wnt/β-catenin pathway activation

**DOI:** 10.1038/s41389-026-00618-0

**Published:** 2026-05-06

**Authors:** Linna Cheng, Huiyang Zhang, Liqun Guo, Qing Zhang, Yuanyuan Zhang, Qi Xie, Shuai Zhou, Dongpeng Wen, Wenchao Chen, Zhikai Wang, Jiancheng Zhang, Rick F. Thorne, Zunmin Zhu

**Affiliations:** 1 Institute of Hematology, Henan Key Laboratory of Stem Cell Clinical Application and Key Technology, People’s Hospital of Zhengzhou University, Henan Provincial People’s Hospital, School of Clinical Medicine, Henan University, Zhengzhou, Henan China; 2 Translational Research Institute, People’s Hospital of Zhengzhou University, Henan Provincial People’s Hospital, School of Clinical Medicine, Henan University, Zhengzhou, Henan China; 3 Department of Pathology, People’s Hospital of Zhengzhou University, Henan Provincial People’s Hospital, School of Clinical Medicine, Henan University, Zhengzhou, Henan China; 4 The First Department of Gastrointestinal Surgery, Henan Provincial Research Center for Precision Control Engineering of Digestive Tract Tumors, People’s Hospital of Zhengzhou University, Henan Provincial People’s Hospital, School of Clinical Medicine, Henan University, Zhengzhou, Henan China

**Keywords:** Colorectal cancer, Cell signalling

## Abstract

Abnormal Wnt/β-catenin pathway activation drives colorectal cancer (CRC) tumorigenesis, yet effective targeted therapies remain elusive. Given HPCAL1’s established dual tumor-suppressive and oncogenic roles in other cancers, this study investigates its function in CRC to assess the therapeutic potential. Bioinformatic analyses of publicly available CRC datasets supported by in-house cohort studies linked high HPCAL1 expression in primary CRC tissues with clinicopathological factors associated with metastasis and worsened patient outcomes. Knockdown and overexpression studies in cell lines showed that HPCAL1 positively contributes to CRC cell motility and invasion, as well as proliferation in vitro and in vivo in xenografts. RNA sequencing linked HPCAL1 expression with the Wnt/β-catenin pathway, demonstrating positive correlations with Wnt ligands in CRC models and clinical samples. Biochemical approaches showed HPCAL1 augmented the activation and nuclear localization of β-catenin. Moreover, HPCAL1 formed distinct complexes with β-catenin in tandem with the TCF7 or p65 transcription factors, in turn, differentially transactivating Wnt6, Wnt7A, and Wnt11 ligands. Notably, the anticancer activity of desloratadine against CRC cells, a pharmacological inhibitor of HPCAL1, functioned by curtailing Wnt6, Wnt7A, and Wnt11 expression and suppressing Wnt/β-catenin signaling. Collectively, these findings indicate that HPCAL1 is a significant contributor to the clinical aggressiveness of CRC with oncogenic effects intrinsically linked with sustaining canonical Wnt pathway activation. Furthermore, drug targeting experiments provide proof-of-principle evidence for promoting HPCAL1 as a therapeutic target for countering activated Wnt/β-catenin signaling in colorectal cancer.

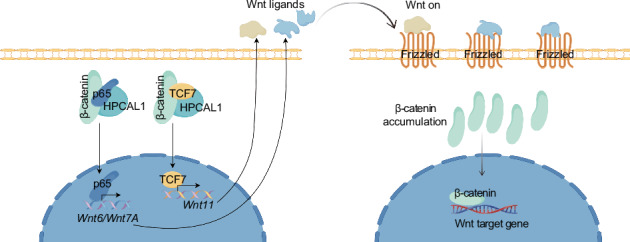

## Introduction

Colorectal cancer (CRC) remains a significant global health issue, being one of the most frequently diagnosed cancers and a leading cause of cancer-related mortality [1]. Despite improvements in early diagnosis and treatment optimization, the clinical management of CRC continues to present significant challenges, highlighting the urgency for alternative therapeutic strategies [2]. This underscores the need to fully comprehend the mechanisms that drive CRC initiation and growth, allowing progress in disease prevention as well as serving to identify effective molecular targets that will aid in developing new interventions.

Early studies in familial CRC uncovered a causal role for mutations in the APC (adenomatous polyposis coli) gene, a core regulator of the Wnt pathway. Stemming from this initial discovery, extensive sequencing of sporadic tumors has uncovered mutations affecting other regulatory molecules associated with Wnt signaling. Indeed, abnormal Wnt activation is thought to be a key driver in the development of various cancers [3], although notably, CRC cases in particular exhibit a remarkable ~80% incidence of mutations in Wnt-pathway associated genes [4]. Commonly, these involve somatic mutations that prevent the degradation and turnover of β-catenin, the key effector of the canonical Wnt pathway. Most frequently, such mutations occur in *APC* along with *AXIN1/2*, although mutations in the β-catenin gene can also render its protein product resistant to degradation [3, 5, 6]. Moreover, the overexpression of various Wnt ligands also reinforces β-catenin stabilization and nuclear translocation, together with the epigenetic silencing and downregulation of Wnt inhibitor genes such as DKK1 (Dickkopf-1) and WIF-1 (Wnt inhibitory factor-1) [3, 6]. On this basis, intensive drug development efforts have focused on targeting Wnt/β-catenin signaling although with mixed results due to the pathway’s inherent complexity as well as toxicity issues associated with different Wnt-targeting agents [7, 8].

Of relevance to this report, hippocalcin-like 1 (HPCAL1) is a neuronal calcium sensor protein, alternatively known as VILIP-3, belonging to the visinin-like protein (VILIP) superfamily [9]. HPCAL1 contributes to calcium-dependent regulation of signal transduction cascades, particularly within the nervous system where its deregulation has been implicated in pathologies including Alzheimer disease and schizophrenia [10]. However, more recent studies have highlighted the diverse role of HPCAL1 among different cancers. On the one hand, HPCAL1 was described to act as a tumor suppressor that variously inhibits cell cycle progression, cell adhesion and migration, the epithelial-mesenchymal transition in esophageal squamous cell carcinoma [11, 12], non-small cell lung carcinoma [13] and hepatocellular carcinoma [14]. Conversely HPCAL1 was reported to facilitate the proliferation of glioblastoma cells in vitro [15] and its elevated expression in cholangiocarcinoma was found to be an indicator of worsened prognosis [16]. Nonetheless, the contextual role of HPCAL1 in the pathological development of CRC remains unknown. Moreover, save for a single associative report in glioblastoma [15], no direct evidence currently exists to show that HPCAL1 influences the Wnt pathway.

This study now shows that HPCAL1 acts as a facilitator of Wnt/β-catenin activation in CRC. Foremost, we demonstrate that HPCAL1 is differentially overexpressed in CRC tissues compared to normal colonic tissues and uncover an association between high HPCAL1 expression and poor patient outcomes. Our in vitro investigations show that HPCAL1 substantially achieves Wnt signaling pathway activation through promoting the expression of Wnt ligands. Mechanistically, this results from HPCAL1 establishing discrete interactions with the TCF7 and p65 transcription factors, acting in concert with β-catenin to specify transcription of downstream Wnt gene targets. Taken together, our findings support a model whereby HPCAL1 promotes CRC aggressiveness by sustaining canonical Wnt signaling, thereby functioning as a critical facilitator of the cellular plasticity required for metastatic progression [17]. The strong diagnostic and prognostic associations observed in clinical specimens further underscore the potential of HPCAL1 as a biomarker and therapeutic target in CRC.

## Materials and methods

### Clinical specimens

Paired colorectal cancer and corresponding adjacent nontumor intestinal tissues were collected during surgical treatment at the First Department of Gastrointestinal Surgery, Henan Provincial People’s Hospital (Zhengzhou, China). Samples were divided for later analysis of mRNA, protein and immunohistochemical staining. Ethical approval for specimen procurement was approved by the Ethics Committee of Henan Provincial People’s Hospital (approval 2021-144). Written informed consent was obtained from all individual participants included in the study.

### Protein and RNA tissue extraction

Protein extracts were prepared from patient tissues using RIPA buffer supplemented with protease and phosphatase inhibitors (TargetMol, Boston, USA). After tissue homogenization (Servicebio, Wuhan, China), the extracts were centrifuged at 12,000 *g* at 4 °C for 10 min and total protein concentrations measured using a BCA protein assay kit (Beyotime Biotechnology, Shanghai, China). Alternatively, total RNA was extracted using Trizol reagent according to the manufacturer’s instructions and RNA concentrations measured using a Nanodrop instrument (Thermo Fisher, USA).

### Immunohistochemical staining

Paired CRC and normal tissues were fixed with 4% formaldehyde and embedded in paraffin blocks. After sectioning, the specimens were deparaffinized using xylene and rehydrated in graded ethanol solutions before antigen repair for 30 min at 37 °C. The sections were blocked with 3% BSA and incubated with the indicated primary antibodies overnight at 4 °C; anti-HPCAL1 (1:500 dilution, Proteintech, IL, USA). Subsequently, sections were stained with anti-rabbit secondary antibodies (1:200 dilution, Servicebio, China), followed by incubation with DAB substrate and counter staining with hematoxylin solution.

### Cell culture

The human intestinal epithelial cell line NCM460 and CRC cell lines were obtained from the Cell Bank of the Chinese Academy of Sciences (Shanghai, China). CRC cell lines and HEK-293T cells were cultured in DMEM medium (Biological Industries, Beit Haemek, Israel) while NCM460 cells were cultured in RPMI 1640 medium (Biological Industries, Beit Haemek, Israel), all supplemented with 10% FBS (Biological Industries, Beit Haemek, Israel). All cells were incubated in a 37 °C humidified incubator with a 5% CO_2_ atmosphere. Cells were confirmed mycoplasma-free and used within 25–30 population doublings post-thawing.

### Plasmids construction and transfection

The shRNAs targeting *HPCAL1*, *TCF7*, and *p65* (Table S1) were synthesized (General Biotech, Anhui, China), cloned into pLKO.1, and cotransfected with psPAX2 and VSV-G/pMD2.G into HEK-293T cells using Lipofectamine 2000 (Thermo Fisher Scientific, Waltham, MA, USA). Viral supernatants harvested 48-72 h post-transfection were used to transduce target cells with 8 μg/mL polybrene (General Biotech, Anhui, China), followed by puromycin selection (1 μg/mL, TargetMol, Boston, USA) 24 h later.

### Immunoprecipitations

Cells were lysed in RIPA buffer supplemented with protease inhibitor cocktail solution (TargetMol, Boston, USA). Lysates were cleared by centrifugation (14,000 rpm, 15 min, 4 °C). Supernatants were incubated overnight at 4 °C with specific antibodies or normal IgG controls. Protein A/G agarose beads (Beyotime Biotechnology, Shanghai, China) were added for 2 h at 4 °C. After RIPA buffer washes, immunoprecipitated complexes were boiled in 2× SDS buffer for western blot.

### Western blotting

Cells were lysed in RIPA buffer (Beyotime Biotechnology, Shanghai, China). Twenty micrograms of total protein per sample was separated by SDS-PAGE and transferred to NC membranes (Millipore, Bedford, MA, USA). Membranes were then blocked and incubated with primary antibodies at 4 °C overnight (listed in Table S2) and further incubated with species-specific secondary antibodies (Abways Technology, Shanghai, China). Proteins were visualized using ECL (Kermey, Zhengzhou, China) and on a Bio-Rad ChemiDoc system.

### qRT-PCR

Total RNA was extracted using Trizol (Solarbio, Beijing, China) and cDNA templates were synthesized using the cDNA Synthesis SuperMix (Kermey, Zhengzhou, China). qRT-PCR reactions were then performed in a StepOne™ real-time PCR system (Thermo Fisher, Waltham, USA) using Universal SYBR Green Supermix (Kermey, Zhengzhou, China). GAPDH was used as the internal reference gene. Specific primer sequences are listed in Table S3.

### Cell viability and proliferation assays

Cell viability was evaluated using the Cell Counting Kit-8 (Targetmol, Boston, USA). Briefly, cells seeded in 96-well plates (1000 cells/well) were cultured for the indicated durations before incubation with 10 μL CCK-8 reagent/well for 2 h. Absorbance was measured at 450 nm. Alternatively, colony formation was conducted by culturing 1000 cells per well in 6-well plates over 10 days. Colonies were then fixed with 4% formaldehyde, stained with 0.1% crystal violet (Beyotime Biotechnology, Shanghai, China), imaged and quantified using ImageJ.

### Cell motility and invasion assays

Wound healing was performed on confluent cell monolayers cultured in 6-well plates after creating a uniform scratch using a sterile pipette tip. After washing with PBS to remove detached cells, cells were maintained in serum-free DMEM. Wound images were captured at indicated time points. Wound closure was quantified by measuring the area recovery, expressed as [(Area at 0 h − Area at 24 h) / Area at 0 h] × 100%, using ImageJ software.

Alternatively, cell migration assays were conducted using Transwells (8 µm, Corning, New York, USA). Briefly, 2 × 10^4^ cells in serum-free medium were seeded into the upper chamber, while the lower chamber contained complete medium. After 24 h incubation, cells that migrated to the underside of the membrane were fixed with 4% formaldehyde (15 min), stained with 0.1% crystal violet, and counted in five random microscopic fields per well.

### In vivo subcutaneous tumor model

To control for inter-animal variability, a paired experimental design was employed. A total of nine 6-week-old female BALB/c nude mice each received subcutaneous injections of both cell types: HCT116-shCtrl cells into the right dorsal flank and HCT116-shHPCAL1 cells into the left dorsal flank (1 × 10⁷ cells per site). Thus, each animal served as its own control (*n* = 9 paired observations). Tumor volumes were measured every two days for two weeks using the formula: V = (shortest diameter)² × longest diameter / 2. Tumors were harvested after two weeks for further analysis. All animal experiments were approved by the Zhengzhou University Animal Care and Use Committee (Approval No.: ZZU-LAC20230526 [23]) and complied with relevant guidelines.

All animals that completed the experimental protocol were included in the analysis. No data points or animals were excluded from the study. The sample size of nine animals was determined based on the effect magnitude observed in preliminary data and the high statistical power inherent to the paired design, which is sufficient to detect a biologically significant difference using a two-tailed paired *t*-test (α = 0.05). To minimize physiological variability and enhance statistical clarity in this foundational study, we used a female-only cohort, which also facilitated social housing and improved animal welfare. Future studies will specifically address potential sex-based differences. The investigator assessing the primary outcomes was blinded to the identity of the injected cell type (shCtrl vs. shHPCAL1) for each flank throughout the experiment and data analysis. Group allocation codes were only revealed after all measurements and statistical analyses were completed.

### Dual luciferase assay

Proximal promoter fragments of *Wnt7A* and *Wnt11* were amplified from HCT116 cell genomic DNA and subcloned into the pGL3-basic luciferase reporter vector. *TCF7* and *p65* were cloned into pCMV-MCS-P2A-mCherry-Myc-Hyg (Beyotime Biotechnology, Shanghai, China). pRL-TK (Beyotime Biotechnology, Shanghai, China) was co-transfected as an internal control to assess transfection efficiency. All vector constructs were verified by DNA sequencing. Dual luciferase assays were conducted on the indicated cells following 24 h transfection with the indicated pGL3 firefly luciferase reporter plasmids in combination with the pRL-TK *Renilla* luciferase plasmid. Luciferase activity was measured using the Dual-Glo® Luciferase Assay System (Promega, WI, USA) according to the manufacturer’s protocol with normalization against *Renilla* luciferase activity. Triplicate wells were used for each sample.

### RNA sequencing and analysis

Total RNA was extracted from HCT116 cells with and without HPCAL1 knockdown using Trizol Reagent (Invitrogen, Carlsbad, CA, USA). RNA quality was assessed by NanoDrop (Thermo Scientific, Waltham, MA, USA). Sequencing libraries were constructed from 3 µg RNA. Briefly, mRNA was enriched with oligo-dT beads, fragmented, reverse transcribed, end-repaired, adapter-ligated, size-selected (400–500 bp), and PCR-enriched (15 cycles).

Library quality was validated on the Bioanalyzer 2100 system (Agilent Technologies, Santa Clara, CA, USA). paired-end 150 bp sequencing was performed on the Illumina NovaSeq 6000 platform at Shanghai Personal Biotechnology Co. Ltd. For analysis, raw reads were trimmed (FastQC, Trimmomatic) and aligned to GRCh38 with STAR. Gene counts were obtained via featureCounts. Differential expression was analyzed using DESeq2 (*p*.adj <0.05, |log2FC | > 1). GO enrichment of DEGs was performed with topGO (hypergeometric test, *p* < 0.05).

### Molecular docking analysis

The structures of HPCAL1 (PDB: 5T7C), β-catenin (PDB: 1G3J), and p65 (RelA) (PDB: 1NFI) were retrieved from the Protein Data Bank. The structure of TCF7 was modeled de novo using SWISS-MODEL. Prior to docking, all protein structures underwent preparation using AutoDockTools-1.5.7 [18]. Protein-protein docking was performed using the GRAMM-X server [19, 20]. Subsequently, the resulting docking poses were further processed with AutoDockTools-1.5.7. Protein-protein interaction analyses were conducted, and complex models were visualized and rendered using PyMOL.

### Bioinformatic analysis

Clinical and gene expression information for colorectal cancer patients included in The Cancer Genome Atlas (TCGA) COAD dataset together with the TCGA Normal dataset from normal colonic tissues were downloaded for offline analyses. Unpaired sample analysis was conducted to delineate gene expression differences between 51 normal tissues and 647 CRC tissues, whereas paired sample analysis was performed on a cohort of 50 patient-matched tumor and adjacent non-tumor samples. The analyses leveraged the R packages (ggplot2 [3.3.6], stats [4.2.1], car [3.1-0]). ROC analysis of the data was performed using the pROC package, and the results were visualized using ggplot2(pROC [1.18.0], ggplot2 [3.3.6]). Survival analysis was performed using the survival package (survival [3.3.1]) and visualized using the survminer and ggplot2 packages (survminer [0.4.9], ggplot2 [3.3.6]).

### Statistical analysis

All measurement data were expressed as the mean ± SD and were analyzed using GraphPad Prism software. For comparisons between two groups, Student’s t-test was used. For comparisons among more than two groups, one-way analysis of variance (ANOVA) was used. *p < 0.05, **p < 0.01, and ***p < 0.001 were considered significant.

## Results

### HPCAL1 is differentially upregulated in CRC and defines clinical aggressiveness

**Fig. 1:**
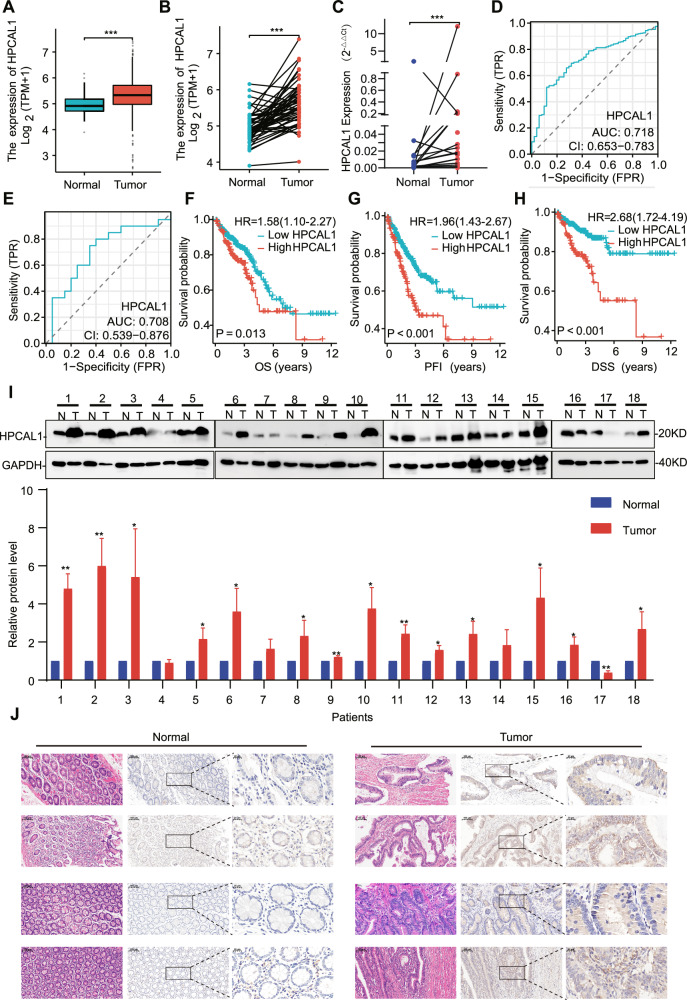
HPCAL1 is upregulated in CRC with high HPCAL1 expression correlating with poor patient outcomes. **A, B** Comparison of HPCAL1 mRNA levels in CRC (*n* = 647) versus paracancerous tissues (*n* = 51, ***, *p* < 0.001) (A) and in matched CRC/normal tissue pairs (*n* = 50 pairs, ***, *P* < 0.001) (B) from the TCGA COAD and Normal datasets. **C** qPCR analysis comparing HPCAL1 mRNA levels from the in house cohort of CRC and adjacent tissue counterparts (*n* = 18 paired tissues, ***, *p* < 0.001). **D**, **E** Diagnostic potential of HPCAL1 expression in CRC. ROC curve plots illustrating the relationship between sensitivity (true positive rate) and specificity (true negative rate) of HPCAL1 expression were calculated from the paired tissue data presented in (B; D) and (C; E), respectively. **F–H** Kaplan-Meier analysis of overall survival (OS; F), progression-free interval (PFI; G) and disease-specific survival (DSS; H) in the TCGA-COAD cohort with patients stratified by median HPCAL1 expression. **I** Western blotting comparing HPCAL1 protein levels in CRC tissues and adjacent counterparts in the cohort from (**C**). GAPDH was used as a loading control. **J** Representative examples of H&E staining and immunohistochemical staining against HPCAL1 in CRC tissues and paracancerous tissues in the cohort from (**C**).

To define the expression status of HPCAL1 in CRC, we undertook offline analyses of the TCGA CRC dataset in an R (v3.2) environment. We observed that HPCAL1 mRNA expression in CRC tumor tissues was significantly higher than in normal colon tissues, and comparisons of patient-matched tissue pairs showed a mostly increased expression trend for individual cases in cancerous versus normal (paracancerous) tissues (Fig. 1A, B). Moreover, our in-house analysis of 18 freshly isolated CRC and adjacent normal tissues reproduced the increased expression trend for HPCAL1 mRNA in CRC versus normal tissues (Fig. 1C). Applying receiver operating characteristic (ROC) analyses to these data indicated that HPCAL1 mRNA expression was able to effectively differentiate between cancerous and normal tissues with an area under the curve (AUC) of 0.718 and 0.708 in the two datasets (Fig. 1D, E). Moreover, the increase in HPCAL1 expression was significantly correlated with N stage, pathologic stage and lymph node metastasis (Table 1). Indeed, suggesting clinical significance, stratification of CRC patients according to tumor HPCAL1 expression showed that higher HPCAL1 expression was associated with significantly worse overall survival (OS), progression-free interval (PFI) and disease-specific survival (DSS) (Fig. 1F–H). Nevertheless, since transcript levels may not reflect protein expression, it was also important to demonstrate that the differential expression status of HPCAL1 mRNA in CRC was reflected at the protein level. In support, immunoblotting analyses comparing cancerous and paracancerous tissues in the in-house CRC cohort showed that HPCAL1 protein levels were predominantly increased in malignant tissues (Fig. 1I). Moreover, the same trend was evident after conducting immunohistochemical staining against HPCAL1 where the strongest staining was predominantly observed within tumor cells (Fig. 1J, Fig. S1A). Single-cell transcriptome analysis of a CRC cohort from the scCancerExplorer database further revealed that HPCAL1 expression was predominantly restricted to malignant cells, with minimal levels in the stromal and immune compartments (Fig. S1B–D). Collectively, these data indicate that HPCAL1 is frequently elevated in CRC cells and further suggest an association between upregulated HPCAL1 expression and clinical aggressiveness.

**Table 1:** Correlation between HPCAL1 levels and clinicopathologic parameters in 644 cases of CRC.

Characteristics	Low expression of HPCAL1	High expression of HPCAL1	*p* value
*n*	322	322	
Pathologic T stage, *n* (%)			0.754
T3&T4	253 (39.5%)	257 (40.1%)	
T1&T2	67 (10.5%)	64 (10%)	
Pathologic N stage, *n* (%)			**0.004**
N1&N2	117 (18.3%)	155 (24.2%)	
N0	201 (31.4%)	167 (26.1%)	
Pathologic M stage, *n* (%)			0.327
M1	39 (6.9%)	50 (8.9%)	
M0	235 (41.7%)	240 (42.6%)	
Pathologic stage, *n* (%)			**0.013**
Stage III&Stage IV	120 (19.3%)	154 (24.7%)	
Stage I&Stage II	188 (30.2%)	161 (25.8%)	
Primary therapy outcome, *n* (%)			0.231
CR	138 (47.4%)	120 (41.2%)	
PD	14 (4.8%)	19 (6.5%)	
Lymphatic invasion, *n* (%)			**<0.001**
Yes	95 (16.3%)	137 (23.5%)	
No	193 (33.2%)	157 (27%)	
Residual tumor, *n* (%)			0.267
R1&R2	16 (3.1%)	26 (5.1%)	
R0	220 (43.1%)	248 (48.6%)	
Perineural invasion, *n* (%)			0.515
Yes	30 (12.8%)	30 (12.8%)	
No	96 (40.9%)	79 (33.6%)	
Colon polyps present, *n* (%)			0.473
Yes	55 (17%)	44 (13.6%)	
No	134 (41.5%)	90 (27.9%)	
History of colon polyps, *n* (%)			0.134
Yes	79 (14.2%)	99 (17.8%)	
No	193 (34.8%)	184 (33.2%)	
CEA level, *n* (%)			0.910
> 5	77 (18.6%)	77 (18.6%)	
<= 5	129 (31.1%)	132 (31.8%)	
Histological type, *n* (%)			**0.006**
Mucinous adenocarcinoma	30 (4.7%)	53 (8.4%)	
Adenocarcinoma	287 (45.3%)	263 (41.5%)	

Note: Values in bold indicate statistical significance（*p* < 0.05).

### HPCAL1 promotes the malignant phenotype of CRC cells

**Fig. 2:**
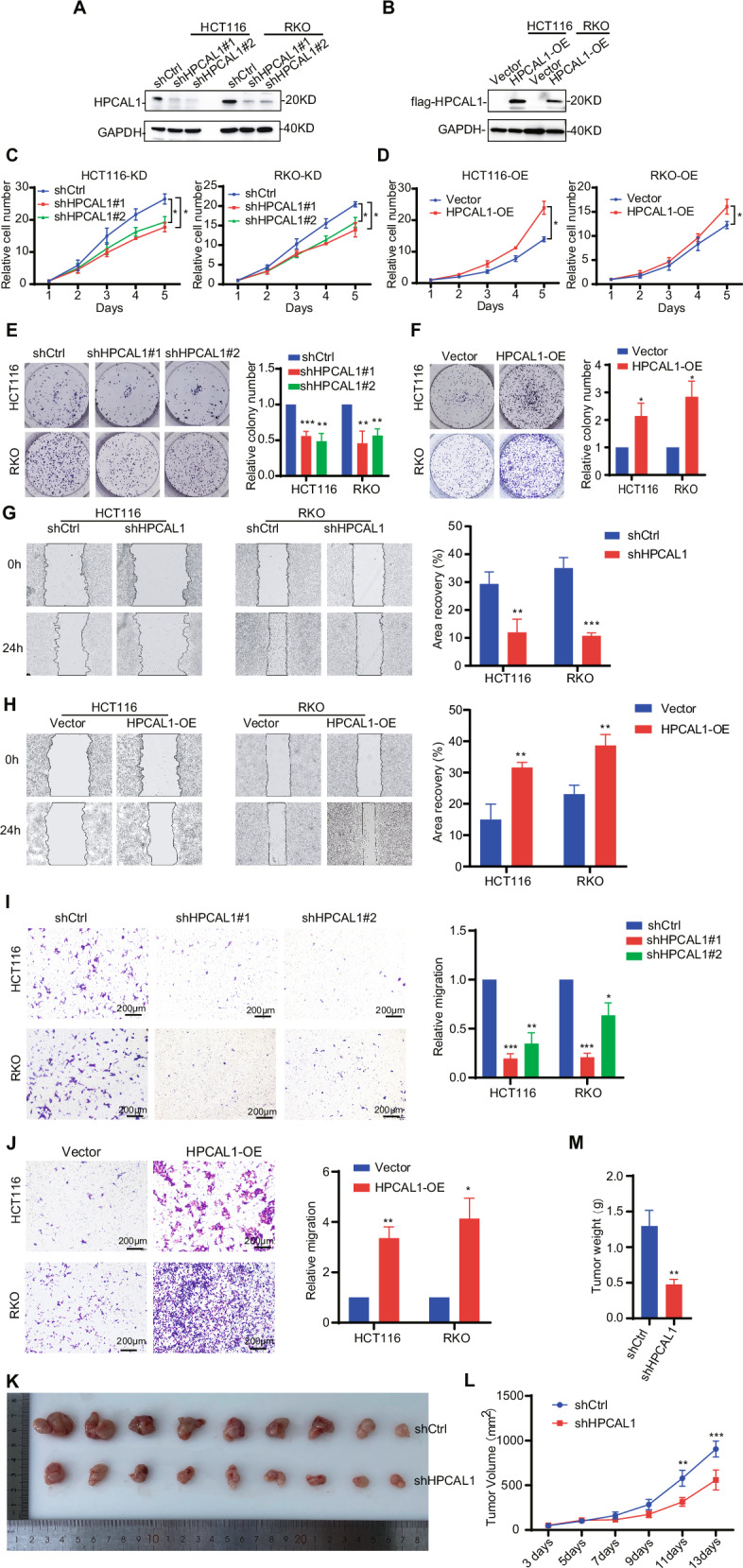
HPCAL1 expression promotes CRC tumorigenic phenotypes in vitro and in vivo. **A**,**B** Western blotting analyses of HPCAL1 protein levels in HCT116 and RKO CRC cells after transduction with lentiviruses packaged with independent shRNAs targeting HPCAL1 (shHPCAL1#1 and #2) or a control shRNA (shCtrl) (**A**) or lentiviruses containing HPCAL1 cDNA for overexpression studies (OE) (**B**). **C, D** GAPDH was used as a loading control. Cell viability measured with CCK-8 assays in the HCT116 and RKO cell lines subjected to knockdown (**C**) and overexpression of HPCAL1 (**D**), respectively. Data were normalized to day 1 readings with values representing mean ± s.d. of at least 3 independent experiments (*p < 0.05). **E, F** Representative well images (left) and quantitation of colonies (right) from colony formation assays conducted on the HPCAL1 knockdown (**E**) and overexpression (**F**) cell lines, respectively (*p < 0.05; **p < 0.01; ***p < 0.001). **G, H** Representative microscopic fields (left) and quantitation of area recovery (right) in wound healing assays conducted on the HPCAL1 knockdown (**G**) and overexpression (**H**) cell lines, respectively (*p < 0.05; **p < 0.01). **I, J** Representative microscopic fields (left) and quantitation of migrating cells (right) in Transwell migration assays conducted on the knockdown (**I**) and overexpression (**J**) cell lines, respectively (*p < 0.05; **p < 0.01; ***p < 0.001). **K–M** Control (shCtrl) and knockdown (shHPCAL1) HCT116 cells were subcutaneously inoculated into nude mice. Tumor volumes were estimated by caliper measurements every second day (**K**). Tumor tissues were excised, photographed (**L**) and weighed (**M**) after 13 days (*n* = 9 tumors per experimental group, **p < 0.01, ***p < 0.01).

To obtain insights into the biological basis of how HPCAL1 influences CRC tumorigenesis, we undertook genetic manipulations in CRC cell lines using lentiviral transduction systems, focusing on the HCT116 and RKO cell lines which express readily detectable endogenous levels of HPCAL1 (Fig. S2A, B). Employing shRNA knockdown (KD) with two independent targeting sequences effectively depleted HPCAL1 expression compared with a negative control (NC) sequence (Fig. 2A), while successful overexpression (OE) of Flag-epitope tagged HPCAL1 was readily demonstrated in both cell lines (Fig. 2B).

After establishing stably infected cell lines, we performed CCK-8 and colony formation assays to ascertain the primary effects of HPCAL1 on growth. In CCK-8 assays, knockdown of HPCAL1 was associated with growth reductions in HCT116 and RKO cells whereas ectopic HPCAL1 expression promoted cell growth (Fig. 2C, D). Consistently, HPCAL1 KD led to a markedly lower number of colonies in colony formation assays (Fig. 2E), in contrast to HPCAL1 OE which resulted in patently increased colony formation (Fig. 2F). In parallel with these experiments, we also assessed the effects of HPCAL1 in cell motility and invasion assays which provide proxy measures of metastatic potential. The results of wound healing assays showed larger wound areas in HPCAL1 KD cells after 24 h (Fig. 2G), indicative that depletion of HPCAL1 retarded cell motility, while OE of HPCAL1 promoted cell motility (Fig. 2H). In addition, the application of the manipulated cell lines to Transwell assays showed that HPCAL1 expression is also associated with the invasive potential of CRC cells (Fig. 2I, J). We further demonstrated that the in vitro changes in CRC cell phenotypes associated with HPCAL1 were translatable to the in vivo setting by employing mouse xenograft models. Here, the HPCAL1 KD or NC HCT116 cells were subcutaneously inoculated into BALB/c nude mice with tumor growth measurements together with final tumor weights confirming that the HPCAL1 KD xenografts grew significantly slower in comparison to the controls (Fig. 2K–M).

Collectively these results indicate that HPCAL1 contributes to the tumorigenicity of CRC cells both in vitro and in vivo, positively regulating cell proliferation and motility, providing a likely basis as to how HPCAL1 influences tumor growth and metastasis in patients. However, the underlying details of the pathways associated with these phenotypes remained to be investigated.

### HPCAL1 activates Wnt signaling in CRC

**Fig. 3:**
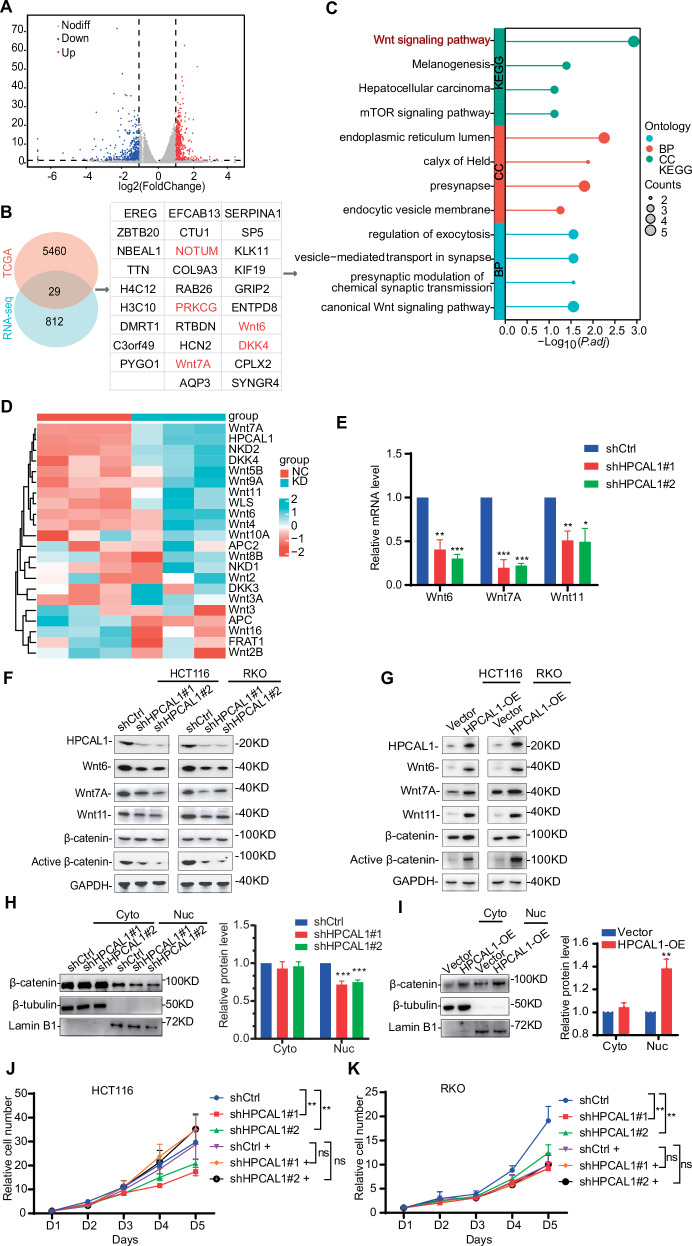
HPCAL1 activates Wnt signaling in CRC. **A** Volcano plot showing downregulated and upregulated genes in HCT116 cells with and without HPCAL1 knockdown using RNA-seq analysis. **B** Venn diagram intersection between the differentially expressed genes from the HCT116 RNA-seq dataset in (**A**) (shHPCAL1 vs shCtrl)and the TCGA-COAD patient cohort(50% low HPCAL1 vs 50% high HPCAL1). The 29 overlapping genes are shown with Wnt pathway-related genes highlighted in red. **C** KEGG and Gene Ontology (GO) analysis of intersecting genes from (**B**) showing significant enrichments for KEGG pathways, cellular component (CC) and biological process (BP) terms. **D** Heatmap illustrating changes in Wnt pathway-related genes in HCT116 cells with and without HPCAL1 knockdown based on the RNA-seq data from (**A**). **E** qPCR analysis of Wnt6, Wnt7A and Wnt11 mRNA level changes in HCT116 cells following HPCAL1 knockdown (*p < 0.05; **p < 0.01; ***p < 0.001). **F, G** Western Blot analysis of Wnt signaling-related proteins after HPCAL1 knockdown (**F**) and overexpression (**G**). GAPDH was used as a loading control. **H, I** Western blot analysis of the cytoplasmic (Cyto) and nuclear (Nuc) distribution of β-catenin in HCT116 cells subjected to HPCAL1 knockdown (**H**) or overexpression (**I**). β-tubulin and lamin B1 were used as markers for the cytoplasmic and nuclear compartments, respectively (*p < 0.05; **p < 0.01; ***p < 0.001). **J, K** HCT116 (**J**) and RKO (**K**) cells bearing a control shRNA or independent shRNAs targeting HPCAL1 knockdown were treated with LiCl. The effects on cell growth were measured using CCK-8 assays (*p < 0.05; **p < 0.01; ***p < 0.001).

We performed RNA-seq analyses on the KD and control isogenic HCT116 cell lines to glean clues regarding the molecular mechanisms engaged by HPCAL1. Defined by cutoff strategies involving |LogFC | >2 & *p*.adj<0.05 values, this analysis yielded 841 differentially expressed genes (DEGs), consisting of 452 significantly upregulated and 389 downregulated genes, respectively (Fig. 3A, Table S4). As a further refinement, we intersected this list with DEGs determined between low and high HPCAL1 expression groups in the TCGA-COADREAD cohort (Fig. 3B, Table S5). The resulting 29-gene set was then used for enrichment analysis to define the common pathways and processes. Notably, the Wnt signaling pathway appeared at the apex of the KEGG list as well as featuring among the top highlighted biological processes (Fig. 3C). On this basis, we used hierarchical clustering to characterize the expression changes in canonical Wnt pathway-related genes in HCT116 cells in response to HPCAL1 modulation. Distinctive groups of Wnt-related genes, particularly Wnt ligands, were found to be either upregulated or downregulated following HPCAL1 knockdown (Fig. 3D). Further accessing TCGA CRC data, we examined if associative relationships existed between HPCAL1 and Wnt ligand expression in CRC tissues. This approach identified significant positive correlations between HPCAL1 and Wnt4, Wnt9A, Wnt5B, Wnt6, and Wnt10A with negative correlations for Wnt3, Wnt8A and Wnt8B (Fig. S3A, B). Similar analysis of an independent CRC dataset (GSE26571) also revealed positive correlations between HPCAL1 expression and that of Wnt7A, Wnt1, Wnt2, Wnt5A and Wnt10B (Fig. S3C, D).

To better elucidate the connection between HPCAL1 and Wnt signaling, we returned to utilize our CRC cell line models. As prospective readouts, we identified that the Wnt6, Wnt7A and Wnt11 proteins were well expressed in HCT116 and RKO cells (Fig. S3E) and importantly, were subject to downregulation at the mRNA level following HPCAL1 silencing (Fig. 3E). As anticipated from these transcriptional changes, the protein levels of Wnt6, Wnt7A and Wnt11 were reduced following HPCAL1 knockdown whereas they were patently increased upon ectopic HPCAL1 expression. Moreover, assessing total and activated (non-phosphorylated Ser45) β-catenin by Western blot indicated that the levels of active β-catenin were reduced by HPCAL1 knockdown while being increased by its overexpression (Fig. 3F, G). Further supporting the notion that HPCAL1 contributes to activation of canonical Wnt signaling, subcellular fractionation studies revealed relative decreases in the nuclear levels of β-catenin in HPCAL1 knockdown CRC cells while increased nuclear β-catenin accompanied HPCAL1 overexpression (Fig. 3H, I). Additional corroborative evidence was provided by activating the Wnt pathway in CRC cells using LiCl [21]. Indeed, LiCl treatment abolished the inhibitory effects of HPCAL1 knockdown on cell growth as measured using CCK-8 (Fig. 3J, K) and clonogenicity assays (Fig. S3F, G).

Together these findings establish an association between HPCAL1 expression and the activation of Wnt signaling with a positive relationship identified between the activation and cellular localization of the core effector β-catenin. However, while the downstream actions of HPCAL1 involve Wnt ligand regulation via transcriptional effects, the nature of the connection between HPCAL1 and canonical Wnt signaling remained to be established.

### Desloratadine suppresses CRC tumorigenesis by targeting HPCAL1 through Wnt signaling

**Fig. 4:**
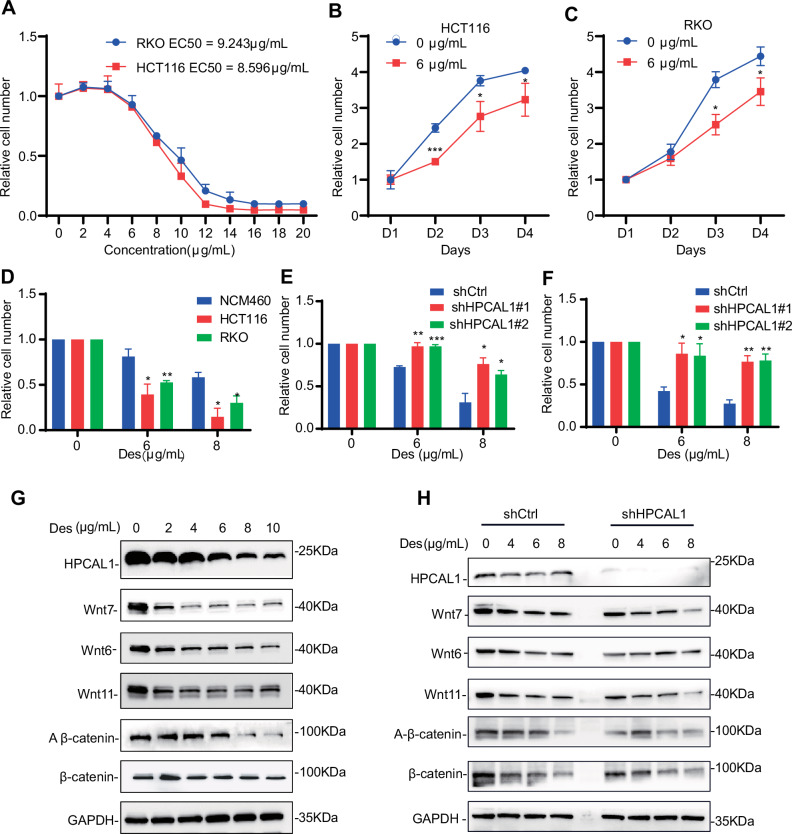
Desloratadine demonstrates anticancer efficacy in CRC by modulating the HPCAL1/Wnt/β-catenin pathway. **A** CCK-8 assays used to determine the half-maximal effective concentration (EC50) of desloratadine against HCT116 and RKO cells after 24 h of treatment. **B, C** HCT116 (**B**) and RKO (**C**) cells were treated without or with desloratadine and cell proliferation measured using CCK-8 assays over 1-4 days (*p < 0.05; ***p < 0.001). **D** Comparative effects of desloratadine against CRC cell lines (HCT116 and RKO) and the normal human intestinal epithelial cell line, NCM460 after 48 h of treatment (*p < 0.05;**p < 0.01). **E, F** Reduction in HPCAL1 expression significantly diminished the antiproliferative impact of desloratadine on cellular growth both in HCT116 (**E**) and RKO (**F**) cells (*p < 0.05; **p < 0.01; ***p < 0.001). **G, H** Dose-dependent effects of desloratadine on the levels of HPCAL1, Wnt ligands and total/activated β-catenin in HCT116 cells measured by Western blotting (**G**). GAPDH was used as a loading control. The assay in (**G**) was repeated to compare the effects of desloratadine on HCT116 cells without and with HPCAL1 knockdown (**H**).

As a corollary to the preceding findings, we considered a recent report involving liver cancer that equated the actions of the repurposed antihistamine drug desloratadine with antagonistic effects against HPCAL1 [22]. This prompted us to examine the actions of desloratadine against CRC and the relationship to the HPCAL1-Wnt axis. As a benchmark experiment, we determined that desloratadine reduced CRC cell viability in a dose- and time-dependent manner with EC50 values of 8.6 and 9.2 µg/mL for HCT116 and RKO cells, respectively (Fig. 4A–C). Moreover, the inhibitory effects of desloratadine were more selective against CRC cells compared with the normal human intestinal epithelial cell line, NCM460 (Fig. 4D). Consistent with these actions involving effects on HPCAL1, the inhibitory effects of desloratadine on cell viability were largely reversed following HPCAL1 knockdown in both HCT116 and RKO cells (Fig. 4E, F). Accordingly, HCT116 and RKO cells engineered to overexpress HPCAL1 exhibited increased sensitivity to desloratadine (Fig. S4). As anticipated, desloratadine treatment resulted in a dose-dependent decline in HPCAL1 levels together with a decline in the expression of Wnt ligands and activated β-catenin (Fig. 4G). Notably, the changes in these markers were not evident in HPCAL1 knockdown cells (Fig. 4H). These experiments suggest that the treatment effects of desloratadine against cancers include downstream effects on Wnt signaling while providing an independent means to confirm the link between HPCAL1 and Wnt signaling.

### HPCAL1 cooperates with TCF7 and p65 to regulate Wnt signaling

**Fig. 5:**
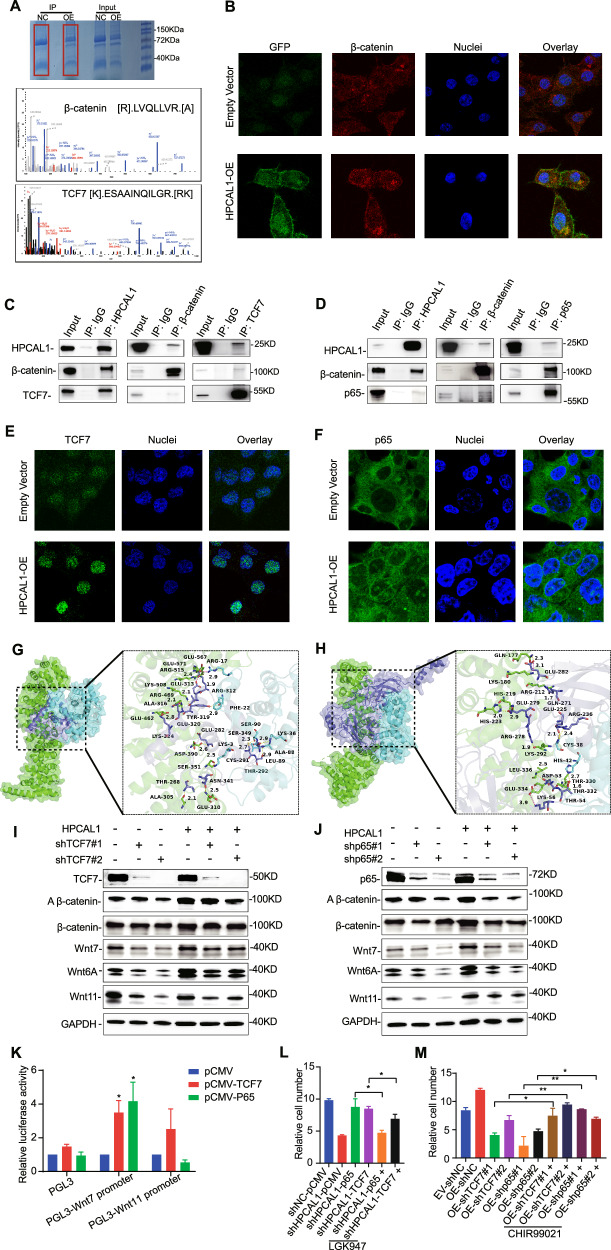
HPCAL1 interacts with β-catenin, TCF7 and p65 to regulate Wnt ligand expression. **A** Coomassie blue stained SDS-PAGE gel showing immunoprecipitates (IP) and input samples prepared from control transfected (NC) and HPCAL1-GFP overexpressing HCT116 cells. The boxed area in red was excised and subjected to mass spectrometry to identify HPCAL1 interacting proteins. Tandem mass spectra of β-catenin and TCF7 are shown. **B** Confocal microscopic images showing colocalization of ectopically expressed HPCAL1 (green) and endogenous β-catenin (red) in RKO cells. Nuclei were counterstained with DAPI (blue). **C** Co-immunoprecipitation analyses conducted in HCT116 cells to measure interactions between HPCAL1, β-catenin and TCF7. Input samples and immunoprecipitates against HPCAL1, β-catenin and TCF7 were subjected to Western blotting as indicated. **D** Co-immunoprecipitation analyses conducted in HCT116 cells to measure interactions between HPCAL1, β-catenin and p65. Input samples and immunoprecipitates against HPCAL1, β-catenin and p65 were subjected to Western blotting as indicated. **E** Ectopic expression of HPCAL1 promotes nuclear localization of TCF7 in RKO cells. Cells were immunostained for TCF7 (green) and counterstained with DAPI (blue) to label nuclei. **F** HPCAL1 expression induces nuclear translocation of p65 in RKO cells. Control cells show diffuse cytoplasmic p65 staining. Immunofluorescence shows p65 (green) and DAPI (blue). **G, H** Complex of HPCAL1/β-catenin/TCF7 (**G**) or HPCAL1/β-catenin/p65 (**H**) predicted using AutoDockTools-1.5.7 and visualized by PyMOL. The TCF7 and p65 proteins are represented as the slate cartoon model, while the HPCAL1 and β-catenin proteins are shown as the cyan and green cartoon models, respectively. The predicted binding sites are shown by the corresponding-colored stick structures. **I, J** Western blotting analysis of Wnt ligands expression in HCT116 cells after knockdown of TCF7 with two independent shRNAs (**I**) or after knockdown of p65 with two independent shRNAs (**J**). GAPDH was used as a loading control. **K** Dual luciferase reporter assays conducted in HCT116 cells comparing pGL3 against vectors containing the proximal promoter regions of the Wnt7 and Wnt11 genes. The cells were co-transfected with empty pCMV vector or with pCMV vectors containing TCF7 and p65 as indicated (*p < 0.05). **L** Cell viability assays in HPCAL1-knockdown HCT116 cells overexpressing Vector, TCF7, or p65, and treated with either DMSO or the Porcupine inhibitor LGK974 (1 μM). Data are presented as mean ± SD (*n* = 3; *p < 0.05;**p < 0.01). **M** Cell viability assays in HPCAL1-overexpressing HCT116 cells transfected with non-targeting control (shNC) or shRNAs against TCF7 or p65, and treated with DMSO or the GSK-3β inhibitor CHIR99021 (5 μM). Data are presented as mean ± SD (*n* = 3; *p < 0.05; **p < 0.01).

We next sought to identify relevant effectors which may connect HPCAL1 to Wnt signaling using mass spectrometric (MS) analysis. Accordingly, we screened candidate proteins that co-immunoprecipitated with ectopically expressed HPCAL1-GFP introduced into HCT116 cells. As anticipated, HPCAL1 was among the 269 protein identities recovered in this analysis along with β-catenin and TCF7 (Transcription Factor 7) (Fig. 5A, and Table S6), the latter known to complex with β-catenin and to engage in Wnt signaling [5, 23–25].

To elaborate on these findings, we assessed the comparative distribution of HPCAL1 and β-catenin using confocal microscopy. Indeed, colocalization was evident between endogenous HPCAL1 and β-catenin, with such staining predominantly evident in the cytoplasmic compartment (Fig. S5A). Strikingly, ectopically expressed HPCAL1 colocalizes with endogenous β-catenin, notably in perinuclear vesicles, providing visual confirmation of their physical proximity (Fig. 5B). We then sought to reconstruct the HPCAL1/β-catenin interaction using the HEK293T transfection model. Consistent with the preceding MS data, endogenous β-catenin was selectively recovered within immunoprecipitates of ectopically expressed HPCAL1-GFP (Fig. S5B). Performing immunoprecipitations against the endogenous proteins in HCT116 CRC cells showed reciprocal interactions were readily detected between HPCAL1 and TCF7, while also confirming that β-catenin interacts with TCF7, at least to some degree (Fig. 5C). A further incidental finding in these experiments involved the NFκB family member p65. Since β-catenin physically interacts with p65 (also known as RELA) [26–28] we used p65 as a positive control to investigate HPCAL1/β-catenin-interactions. Instructively, we confirmed that p65 bound to β-catenin, but with the further unexpected finding that HPCAL1 was reciprocally recovered with p65 (Fig. 5D). Furthermore, ectopic expression of HPCAL1 induced a clear increase in the nuclear localization of TCF7 (Fig. 5E). This directly links HPCAL1 to the regulation of a key transcriptional effector. Similarly, HPCAL1 expression induced distinct nuclear speckling of p65, which was otherwise cytosolic in control cells (Fig. 5F), indicating it promotes NF-κB pathway activation. Independent of these data, we constructed docking models to assess the likely conformation of complexes involving HPCAL1 with β-catenin/TCF7 and with β-catenin/p65 (Fig. 5G, H). Indeed, these models predict that TCF7 and p65 localize to the same pocket of the HPCAL1/β-catenin complex, suggesting a physical basis that underpins the selectivity of their respective interactions. Collectively, these results demonstrate that HPCAL1 activates Wnt/β-catenin signaling by recruiting TCF7 and p65 to form transcriptional complexes that confer precision in the transactivation of Wnt ligand genes. This mechanism aligns with established cooperative roles for TCF7 and p65 in Wnt signaling [25, 29, 30].

To investigate the functional significance of this precision, we first depleted TCF7 levels in HCT116 cells using independent shRNAs while also comparing the effects of HPCAL1 overexpression. As expected, ectopic HPCAL1 expression activated β-catenin, and this was notably unaffected by TCF7 silencing (Fig. 5I). TCF7 knockdown alone reduced endogenous Wnt7A, Wnt6, and Wnt11 levels. However, upon HPCAL1 overexpression, Wnt7A and Wnt6 expression were preferentially restored despite TCF7 knockdown, whereas Wnt11 level remained low. Parallel experiments targeting p65 using independent shRNAs revealed the opposite pattern with p65 depletion largely failing to diminish HPCAL1-driven elevations in Wnt11 expression, while Wnt7 and Wnt6A levels were more strongly affected (Fig. 5J). Thus, the effects of HPCAL1 on Wnt ligand transactivation appear to be selective. This notion was supported by the results of transcriptional reporter assays comparing the actions of TCF7 and p65. Using luciferase reporter plasmids based on the proximal promoter regions of either the Wnt7 or Wnt11 genes, we observed that ectopic expression of either TCF7 or p65 increased Wnt7 promoter activity while TCF7 but not p65 significantly increased Wnt11 activity (Fig. 5K).

To functionally establish that HPCAL1 exerts its effects on cell proliferation through the Wnt pathway by engaging with TCF7 or p65, we performed a series of rescue experiments using pharmacological Wnt modulators. First, we investigated whether the oncogenic phenotypes driven by TCF7 or p65 overexpression in an HPCAL1-deficient background remained dependent on upstream Wnt signaling. The enhanced cell viability and proliferation resulting from overexpression of either TCF7 or p65 in HPCAL1-knockdown cells were markedly suppressed by the Porcupine inhibitor LGK974, which blocks Wnt ligand secretion (Fig. 5L, S5C). Conversely, we asked whether direct activation of Wnt signaling could compensate for the loss of either interaction partner. In HPCAL1-overexpressing cells, knockdown of either TCF7 or p65 significantly impaired cell viability and proliferation. Importantly, these deficits were effectively rescued by treatment with the GSK-3β inhibitor CHIR99021, a potent activator of β-catenin-dependent signaling (Fig. 5M, S5D). This suggests that the functional consequences of disrupting either the HPCAL1-TCF7 or the HPCAL1-p65 interaction can be overcome by downstream Wnt pathway activation. This also indicates that the pro-proliferative effects of both TCF7 and p65, when uncoupled from HPCAL1 regulation, are still contingent upon an active Wnt pathway.

Collectively, these data provide a multi-layered validation of the functional relationship between HPCAL1 and its two interaction partners. The physical interactions, selective effects on Wnt ligand expression, and the reciprocal rescue of proliferation phenotypes with pathway-specific modulators converge to demonstrate that HPCAL1 regulates Wnt signaling-dependent cell proliferation through its interactions with TCF7 and p65. This positions it as a key node that integrates these two regulators to modulate the pathway output.

### Clinical evidence for the HPCAL1-Wnt signaling axis in CRC

Following the established associations between HPCAL1, lymph node metastasis and patient outcomes, we further investigated the connection between HPCAL1 and Wnt signaling in clinical samples. Initial analysis of CRC tissues from the in-house cohort showed significant positive correlations between HPCAL1 mRNA levels and those of several Wnt ligands, including Wnt6, Wnt7A, and Wnt11 (Table 2). To substantiate these findings at a larger scale, we analyzed the TCGA cohort, which confirmed a specific and coordinated transcriptomic relationship among components of the HPCAL1/TCF7/p65 axis and the same set of Wnt ligands (Fig. S6). Together, these results validate core components of the axis at the transcriptomic level, while the selective correlation pattern underscores the post-translational complexity of the regulatory mechanism. This provides general support for the notion that HPCAL1 contributes to Wnt activation in CRC tissues via elevation of Wnt ligand expression.

**Table 2:** Correlation between HPCAL1 and Wnt6, Wnt7A, Wnt11 levels in 18 CRC tissues from the in-house cohort.

id	correlationpearson	*p* value pearson	*p*adj pearson	correlation spearman	*p* value spearman	*p*.adj spearman
HPCAL1	1	0	0	1	0	0
Wnt6	0.56	0.0004	0.0007	0.59	0.0002	0.0004
Wnt11	0.53	0.0008	0.0011	0.53	0.0012	0.0015
Wnt7A	0.48	0.0033	0.0033	0.49	0.0027	0.0027

**Fig. 6:**
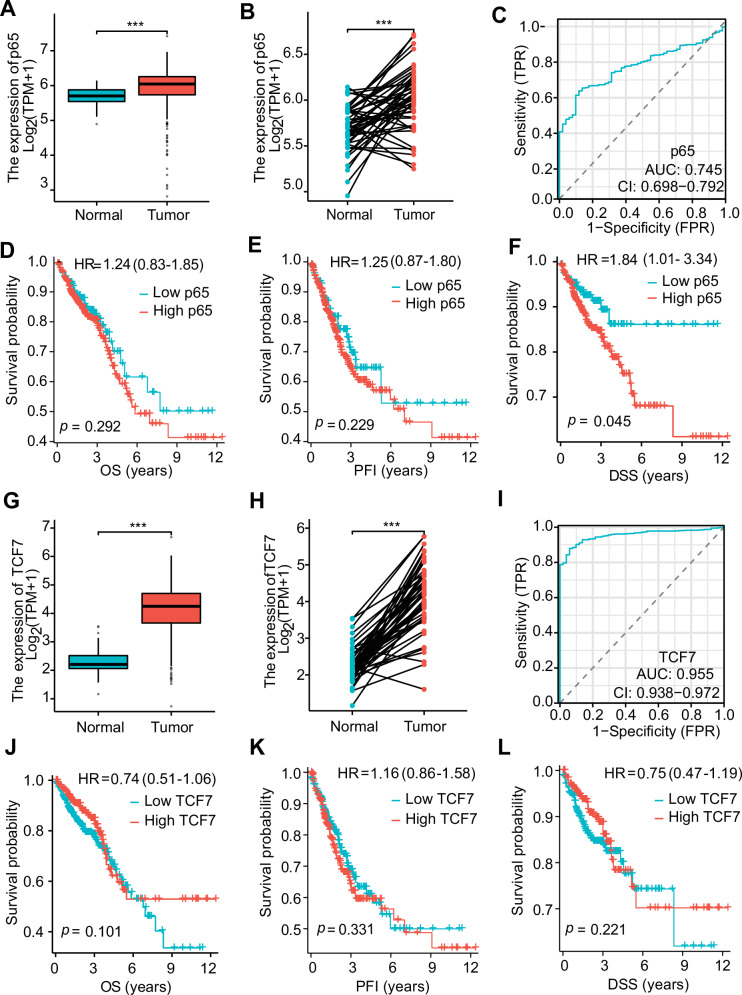
Diagnostic potential and prognostic significance of p65 and TCF7 in CRC. **A, B** Comparison of p65 mRNA levels in CRC (*n* = 647) versus paracancerous tissues (*n* = 51, ***p < 0.001) (**A**) and in matched CRC/normal tissue pairs (*n* = 50 pairs, ***p < 0.001) (**B**) from the TCGA COAD and TCGA Normal datasets. **C** Diagnostic potential of p65 expression in CRC determined using ROC curve analysis calculated from the paired tissue data presented in (**B**). **D-F** Kaplan-Meier analysis of OS (**D**), PFI (**E**) and DSS (**F**) in the TCGA-COAD cohort with patients stratified by median p65 expression. **G, H** Comparison of TCF7 mRNA levels in CRC (*n* = 647) versus paracancerous tissues (*n* = 51,***p < 0.001) (**G**) and in matched CRC/normal tissue pairs (*n* = 50 pairs, ***p < 0.001) (**H**) from the TCGA COAD and TCGA Normal datasets. **I** Diagnostic potential of TCF7 expression in CRC determined using ROC curve analysis calculated from the paired tissue data presented in (**H**). **J-L** Kaplan-Meier analysis of OS (**J**), PFI (**K**) and DSS (**L**) in the TCGA-COAD cohort with patients stratified by median TCF7 expression.

Second, we compared the prognostic value of HPCAL1 against that of p65 and TCF7 in CRC. Akin to HPCAL1 (Fig. 1A), p65 was also differentially overexpressed in CRC tissues versus normal counterparts, with ROC analyses indicating strong diagnostic performance (AUC = 0.745; Fig. 6A–C). Moreover, multivariate analyses showed that increased p65 expression in CRC was significantly correlated with various clinical factors, including pathological N and M stages, the overall pathological stage, lymph node metastasis, perineural invasion, and histological type (Table 3). However, stratification of CRC patients based on p65 expression demonstrated a significant association between elevated p65 expression and worsened DSS, although there were no significant associations detected between OS or PFI (Fig. 6D–F). The expression of TCF7 was even more highly upregulated in CRC tissues compared to either HPCAL1 or P65, showing remarkable performance in ROC analyses (AUC = 0.955; Fig. 6G–I). Additional multivariate analyses revealed that elevated TCF7 expression was significantly associated with several key clinicopathological features, including advanced pathological N and M stages, colon polyp history, and specific histological subtypes (Table 4). Nevertheless, despite the indications of TCF7 as a diagnostic biomarker, no significant associations were determined in clinical outcomes of DSS, OS or PFI (Fig. 6J—L).

**Table 3:** Correlation between p65 levels and clinicopathologic parameters in 644 cases of CRC.

Characteristics	Low expression of p65	High expression of p65	*p* value
*n*	322	322	
Pathologic T stage, *n* (%)			0.367
T3&T4	250 (39%)	260 (40.6%)	
T1&T2	70 (10.9%)	61 (9.5%)	
Pathologic N stage, *n* (%)			**0.008**
N1&N2	119 (18.6%)	153 (23.9%)	
N0	200 (31.2%)	168 (26.2%)	
Pathologic M stage, *n* (%)			**0.009**
M1	32 (5.7%)	57 (10.1%)	
M0	242 (42.9%)	233 (41.3%)	
Pathologic stage, *n* (%)			**0.001**
Stage III&Stage IV	116 (18.6%)	158 (25.4%)	
Stage I&Stage II	193 (31%)	156 (25%)	
Primary therapy outcome, *n* (%)			0.537
CR	132 (45.4%)	126 (43.3%)	
PD	15 (5.2%)	18 (6.2%)	
Lymphatic invasion, *n* (%)			**< 0.001**
Yes	96 (16.5%)	136 (23.4%)	
No	194 (33.3%)	156 (26.8%)	
Residual tumor, *n* (%)			0.347
R1&R2	18 (3.5%)	24 (4.7%)	
R0	236 (46.3%)	232 (45.5%)	
Perineural invasion, *n* (%)			**0.012**
Yes	20 (8.5%)	40 (17%)	
No	91 (38.7%)	84 (35.7%)	
Colon polyps present, *n* (%)			0.902
Yes	52 (16.1%)	47 (14.6%)	
No	116 (35.9%)	108 (33.4%)	
History of colon polyps, *n* (%)			0.230
Yes	98 (17.7%)	80 (14.4%)	
No	187 (33.7%)	190 (34.2%)	
CEA level, *n* (%)			0.190
> 5	70 (16.9%)	84 (20.2%)	
<= 5	136 (32.8%)	125 (30.1%)	
Histological type, *n* (%)			**0.003**
Mucinous adenocarcinoma	54 (8.5%)	29 (4.6%)	
Adenocarcinoma	260 (41.1%)	290 (45.8%)	

Note: Values in bold indicate statistical significance (*p* < 0.05).

**Table 4:** Correlation between TCF7 levels and clinicopathologic parameters in 644 cases of CRC.

Characteristics	Low expression of TCF7	High expression of TCF7	*p* value
*n*	322	322	
Pathologic T stage, *n* (%)			0.610
T3&T4	258 (40.2%)	252 (39.3%)	
T1&T2	63 (9.8%)	68 (10.6%)	
Pathologic N stage, *n* (%)			**0.032**
N1&N2	123 (19.2%)	149 (23.3%)	
N0	198 (30.9%)	170 (26.6%)	
Pathologic M stage, *n* (%)			**0.014**
M1	34 (6%)	55 (9.8%)	
M0	249 (44.1%)	226 (40.1%)	
Pathologic stage, *n* (%)			0.053
Stage III&Stage IV	127 (20.4%)	147 (23.6%)	
Stage I&Stage II	189 (30.3%)	160 (25.7%)	
Primary therapy outcome, *n* (%)			0.678
CR	135 (46.4%)	123 (42.3%)	
PD	16 (5.5%)	17 (5.8%)	
Lymphatic invasion, *n* (%)			0.840
Yes	118 (20.3%)	114 (19.6%)	
No	181 (31.1%)	169 (29%)	
Residual tumor, *n* (%)			0.422
R1&R2	19 (3.7%)	23 (4.5%)	
R0	242 (47.5%)	226 (44.3%)	
Perineural invasion, *n* (%)			0.924
Yes	27 (11.5%)	33 (14%)	
No	80 (34%)	95 (40.4%)	
Colon polyps present, *n* (%)			0.847
Yes	48 (14.9%)	51 (15.8%)	
No	106 (32.8%)	118 (36.5%)	
History of colon polyps, *n* (%)			**0.001**
Yes	107 (19.3%)	71 (12.8%)	
No	172 (31%)	205 (36.9%)	
CEA level, *n* (%)			0.774
<= 5	136 (32.8%)	125 (30.1%)	
> 5	78 (18.8%)	76 (18.3%)	
Histological type, *n* (%)			**<0.001**
Mucinous adenocarcinoma	69 (10.9%)	14 (2.2%)	
Adenocarcinoma	248 (39.2%)	302 (47.7%)	

Note: Values in bold indicate statistical significance (*p* < 0.05).

## Discussion

Both the canonical (β-catenin dependent) and non-canonical (β-catenin-independent) arms of the Wnt signalling pathway are frequently activated in CRC, contributing to CRC development and progression [31]. Arguably, canonical Wnt signalling is more commonly associated with CRC where mutations in key pathway genes are prominent drivers of its activation. However, intensive research efforts have revealed that multifactorial mechanisms are in play, for example, despite the presence of APC or β-catenin mutations [5], colon cancer cells remain responsive to Wnt ligands, indicating a continuing dependence between Wnt ligand expression and downstream signaling [6]. Moreover, alterations in other pathways can also intersect with Wnt to impact β-catenin activity such as the TGF-β signalling axis where loss of SMAD3/4 promotes nuclear β-catenin [32, 33] while the PI3K/AKT/mTOR pathway can also elicit similar actions [34, 35]. This report now adds to this body of literature, positioning HPCAL1 as a facilitator of Wnt/β-catenin signalling in CRC, acting to promote Wnt ligand production and reinforce β-catenin activation. Compelling evidence for this conclusion is provided by several different experimental approaches.

First, β-catenin localization and activation were altered in response to HPCAL1 modulation, providing primary indications that HPCAL1 is associated with the Wnt pathway in CRC. Moreover, manipulating HPCAL1 in CRC cells also resulted in transcriptome changes aligning with effects on Wnt signaling. The notion of a key role for HPCAL1 is further supported by the identification of interactions between HPCAL1 and the Wnt pathway-associated transcription factors TCF7 and p65 [25, 29, 30] as well as with β-catenin itself. Further functional insights were provided where TCF7 and p65 exhibited distinct regulatory impacts on specific Wnt ligands, effects which were confirmed to result from their selective transcriptional activities in concert with HPCAL1. Together, these data reinforce the notion that HPCAL1 modulates Wnt signaling through specific protein complexes in a nuanced manner, underscoring the possible significance of HPCAL1 as a potential therapeutic target, for instance to inhibit Wnt ligand production.

Wnt ligands are secreted into the extracellular milieu and function by engaging the FZD and LRP5/6 coreceptors with the ensuing activation of the Wnt cascade [36]. A range of studies have highlighted the involvement of Wnt ligands in the progression and development of different types of cancer, including colorectal, breast, lung, and endometrial cancer, with specific reports elaborating on the specific actions of Wnt1 [37], Wnt2 [38], Wnt3 [6], Wnt6 [39], Wnt7 [40], Wnt8 [41], Wnt10 [42] and Wnt11 [43], respectively. The clinical significance of elevated Wnt ligand expression is borne out by the efforts to develop therapeutics, specifically to target Porcupine which is an essential enzyme required for Wnt ligand secretion [44, 45]. Several small molecule agents have emerged beyond preclinical development with ongoing early-stage clinical trials [46]. However, one of the inherent problems associated with Porcupine inhibitors is the manifestation of on-target toxicity resulting from the involvement of Wnt in normal tissue homeostasis [47]. Furthermore, the same problem is evident with β-catenin inhibitors, for example, the small-molecule inhibitor PRI-724 that disrupts interactions between β-catenin and its transcriptional co-activators [48]. Thus, targeting HPCAL1 potentially offers an alternative means to counter excessive Wnt ligand production in CRC with reduced toxicity. Indeed, our experiments with desloratadine, a well-studied orally bioavailable drug claimed to target HPCAL1 [22], demonstrated HPCAL1-dependent antiproliferative effects against CRC cells along with diminished production of Wnt ligands and β-catenin activation. These promising findings warrant further exploration of desloratadine repurposing both in CRC as well as other Wnt-dependent cancers.

A caveat to the application of HPCAL1 as a therapeutic target concerns its importance to the etiology of CRC. Here, we show that not only is HPCAL1 mRNA and protein overexpressed in CRC tissues but also that tumor stratification using HPCAL1 provides significant prognostic information. Indeed, multivariate analyses showed high HPCAL1 expression in primary CRC cases was associated with metastasis and delineated poor outcomes for patients. Moreover, positive correlations exist between HPCAL1 and various Wnt ligands, supporting the existence of the HPCAL1-Wnt signaling nexus in CRC tissues. It is also interesting to compare the clinical characteristics of HPCAL1 against its cooperating partner proteins, p65 and TCF7. All three proteins are differentially upregulated in CRC, each individually providing diagnostic information, particularly TCF7 which produced an exceptional ROC score. However, from the perspective of patient outcomes, HPCAL1 expression provided significant stratification for all three survival (OS, DSS and PFI) measures with high hazard ratio ranges. In comparison, while both p65 and TCF7 provided significant correlations against clinicopathological features associated with metastasis, p65 expression was significantly correlated with only DSS while TCF7 expression did not provide useful prognostic information. Thus, while the diagnostic performance of HPCAL1 may not be exemplary, there is no doubt that HPCAL1 expression provides prognostic information that could enhance risk stratification and personalized treatment decisions.

Lastly, it is important to note that while our investigations focussed on associations with Wnt ligands and effects on β-catenin, our transcriptome sequencing data point to other likely effects that HPCAL1 exerts to modulate the Wnt pathway. For example, we observed the downregulation and upregulation of the DKK3/DKK4 and FRAT1 gene expression, which are negative and positive Wnt pathway regulators, respectively [49–53]. However, this contrasts with the upregulation of APC which would be expected to accelerate β-catenin destruction. Nonetheless, the significance of these intriguing findings awaits further experimental validation.

It is noteworthy that our findings which establish an oncogenic role for HPCAL1 in CRC appear to contrast with reports attributing tumor-suppressive functions to it in other malignancies, such as hepatocellular and esophageal cancers [12, 14]. This context-dependent duality may be explained by several mechanisms. In the Wnt-driven milieu of CRC, HPCAL1 is co-opted into a pro-tumorigenic complex with β-catenin, TCF7, and p65. In other tissues, however, it may engage distinct protein interactors, such as those stabilizing the cell cycle inhibitor p21, leading to growth inhibition [14]. Additionally, the potential for functionally divergent isoforms or tissue-specific epigenetic landscapes could further steer its biological output. Thus, HPCAL1 appears to function not as a canonical oncogene or tumor suppressor, but as a pleiotropic signaling adaptor whose role is defined by the cellular context.

## Supplementary information

Supplementary Figures

Supplementary Tables

## Data Availability

All data supporting the findings of this study are available within the article and its supplementary information files, or from the corresponding author upon reasonable request.
