# Supplementary material for: HPCAL1 promotes colorectal cancer progression via TCF7/p65-mediated Wnt ligand upregulation and Wnt/β-catenin pathway activation
Source: Oncogenesis. 2026 May 6;15(1):32. doi: 10.1038/s41389-026-00618-0 (PMC13316108; doi:10.1038/s41389-026-00618-0)
Supplement: Supplementary file 1 — Supplementary Figures [file 41389_2026_618_MOESM1_ESM.pdf]

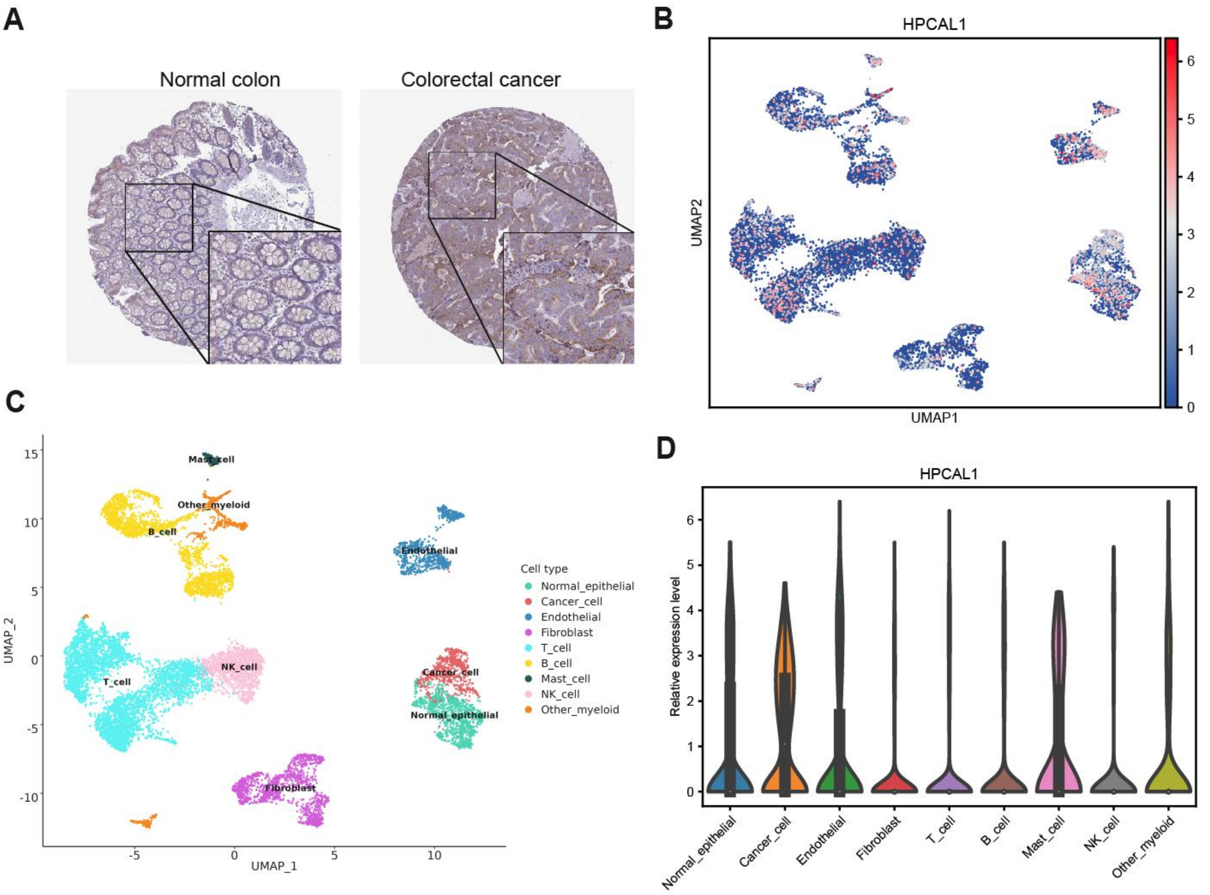

Fig. S1 Expression of HPCAL1 in CRC tissues.

(A) Representative immunohistochemical staining of HPCAL1 in CRC and matched adjacent normal tissues from the Proteinatlas database (https://www.proteinatlas.org/). **(B-D)** Single-cell transcriptome analysis from the scCancerExplorer database (https://www.bianlab.cn/scCancerExplorer/) depicting HPCAL1 expression across major cellular compartments in the CRC tumor microenvironment.HPCAL1 expression in UMAP clusters (B), cell type definitions of UMAP clusters (C) and relative HPCAL1 expression in each cluster (D).

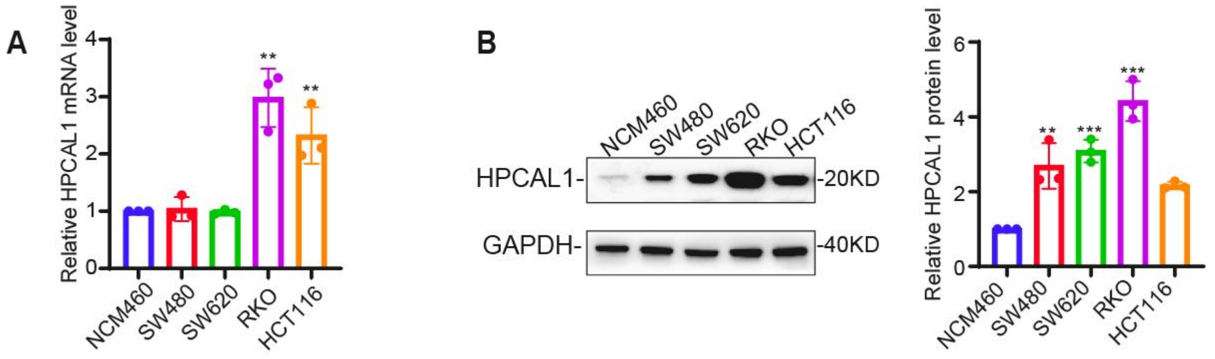

Fig. S2 Expression of HPCAL1 in CRC cell lines.

(A, B) Comparisons of HPCAL1 mRNA (A) and protein (B) levels in the normal colonic epithelial cell line (NCM460) and different CRC (SW480, SW620, RKO and HCT116) cell lines (**, $p < 0.01$; ***, $p < 0.001$).

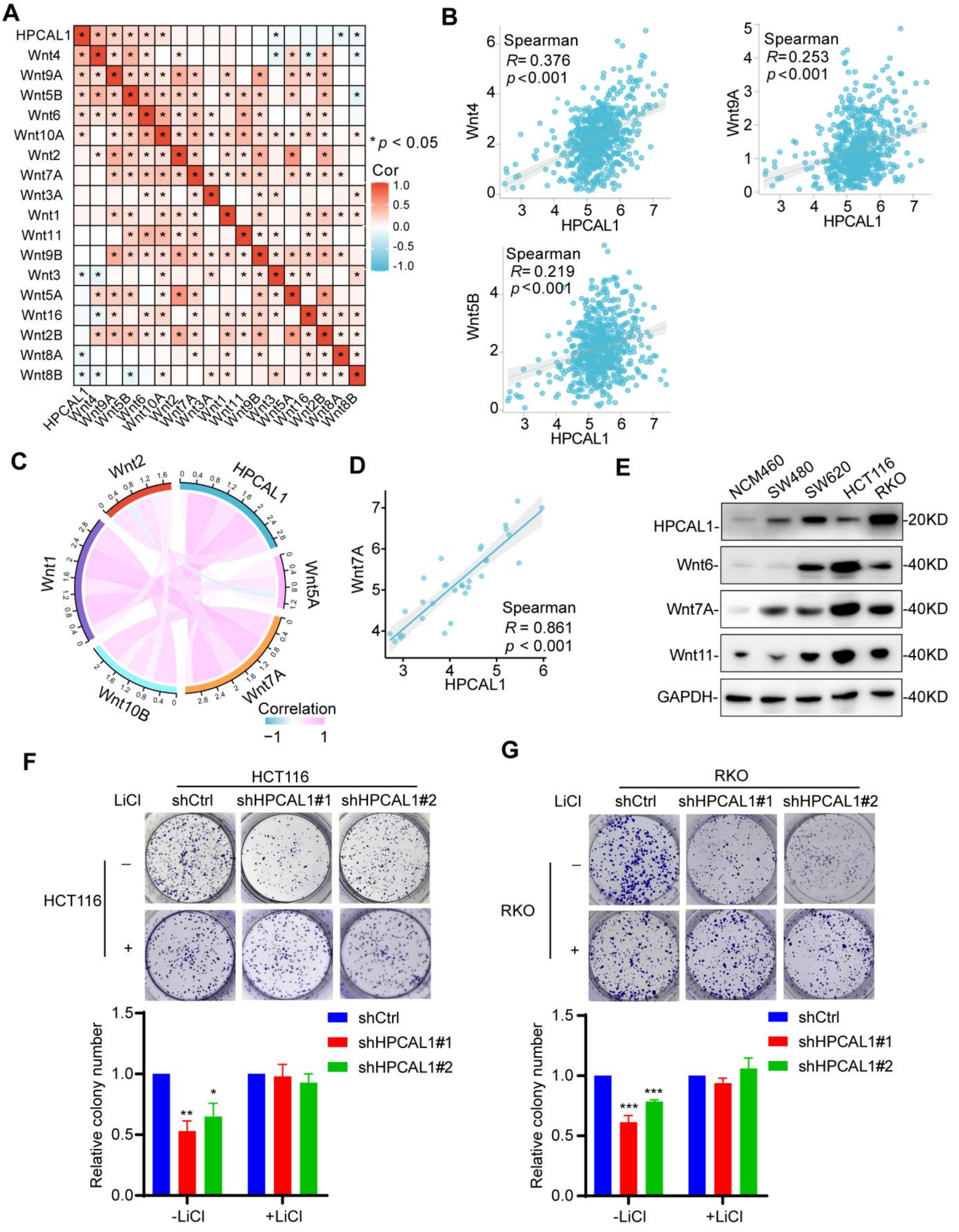

Fig. S3 Correlation analysis and functional effects of HPCAL1 and WNT ligands in CRC.

(A, B) Correlation matrix heatmap of HPCAL1 and Wnt ligands in the TCGA-COAD dataset (A). HPCAL1 expression correlated positively with Wnt4, Wnt9A, Wnt5B, and Wnt6 (all $p < 0.001$) (B). **(C, D)** Circos plot analysis of the GSE26571 CRC cohort (n=12) (C). A strong positive correlation

was observed between HPCAL1 and Wnt7A (R = 0.861, p < 0.001) (D). **(E)** Western blotting analysis of protein levels of HPCAL1, Wnt6, Wnt7A, and Wnt11 in CRC cell lines and the normal colonic epithelial cell line (NCM460). GAPDH was used as a loading control. **(F, G)** HCT116 (F) and RKO (G) cells bearing a control shRNA or independent shRNAs targeting HPCAL1 were treated with LiCl. The effects on cell growth were measured using colony formation assays (*, $p < 0.05$; **, $p < 0.01$; ***, $p < 0.001$).

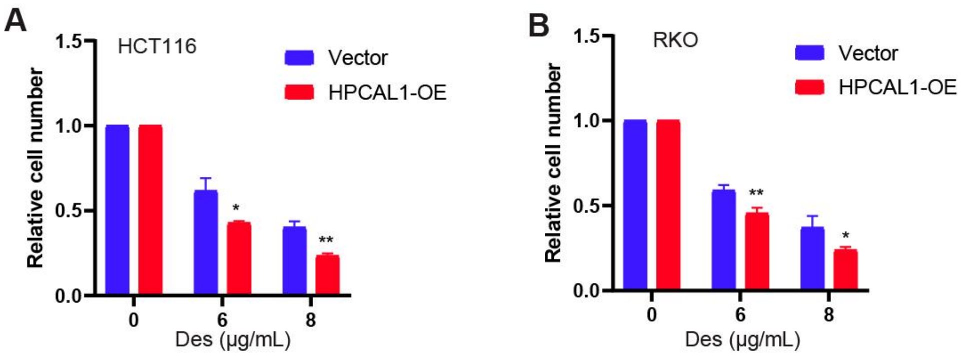

Fig. S4 Forced expression of HPCAL1 sensitizes CRC cells to desloratadine.

Viability of HCT116 (A) and RKO (B) cells with HPCAL1 overexpression after treatment with desloratadine, as determined by CCK-8 assay (*, $p < 0.05$; **, $p < 0.01$).

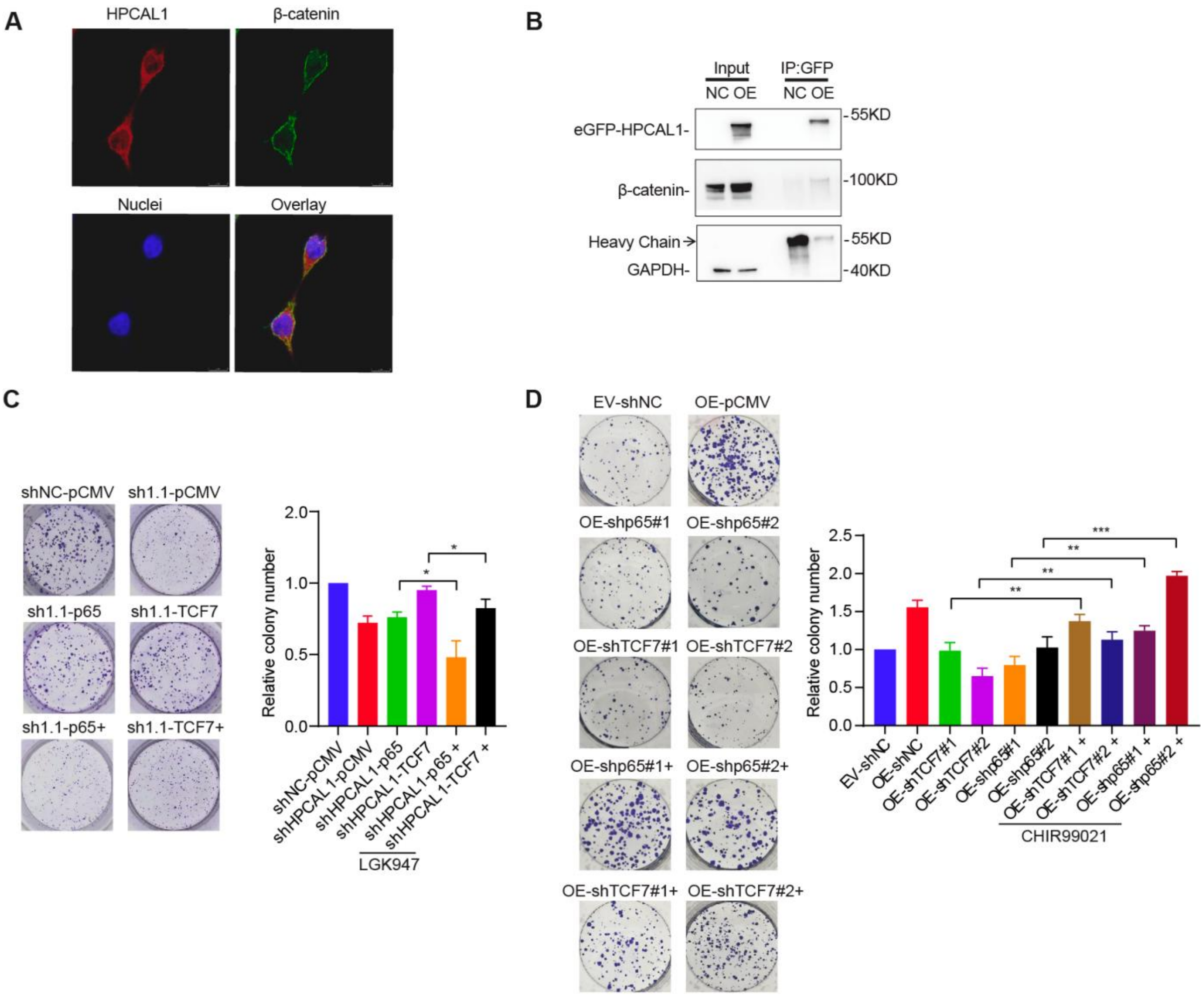

Fig. S5 HPCAL1 associates with β-catenin and regulates colony formation through Wnt/β-catenin signaling.

(A) Confocal microscopic images depicting colocalization between endogenous HPCAL1 (green) and β-catenin (red) in the cytoplasmic compartment of HCT116 cells. Nuclei were counterstained with DAPI (blue). **(B)** Co-immunoprecipitation analyses conducted in control (NC) or HPCAL1-GFP transfected (OE) HEK293T cells. Input samples and GFP immunoprecipitates were subjected to Western blotting against GFP and β-catenin to detect ectopic HPCAL-GFP and endogenous β-catenin, respectively. **(C)** LGK974 treatment abrogates the enhanced colony formation driven by TCF7 or p65 in HPCAL1-knockdown cells (*, $p < 0.05$). **(D)** CHIR99021 treatment restores the impaired colony formation capacity upon TCF7 or p65 knockdown in HPCAL1-overexpressing cells (**, $p < 0.01$; ***, $p < 0.001$).

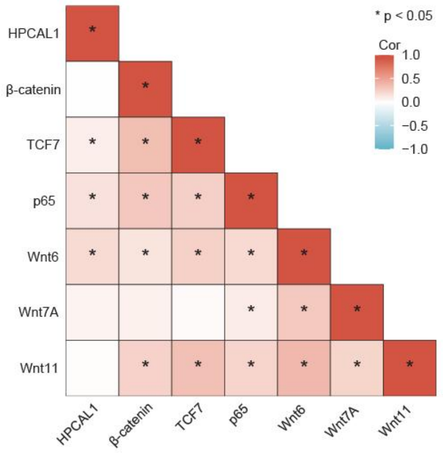

Fig. S6 Correlation analysis linking the HPCAL1/TCF7/p65 axis with Wnt6, Wnt7A, and Wnt11 expression in the TCGA-COAD cohort.
